# A unique approach to compensation and its verification

**DOI:** 10.1186/1476-9255-12-S1-O4

**Published:** 2015-04-16

**Authors:** Ian Dimmick

**Affiliations:** 1 Faculty of Medical Sciences Flow Cytometry Core Facility, Newcastle upon Tyne University, Newcastle upon Tyne, UK

## 

The compensation of multiple fluorochromes within the same experiment enables the correct characterization of cell phenotype by the exclusion of any errors associated with fluorochrome spectral overlap. Fluorochrome compensation is far from standardized, from laboratory to laboratory the reagents and rationale to achieve compensation values varies considerably, so an approach is given to standardize the set up procedure, outlining the major reasons for the steps employed. The first step is to recognize that photomultiplier voltages are of equal importance as the compensation controls to achieve correct fluorochrome compensation, therefore we need to adjust the voltages very carefully to minimize amplification of overlapping fluorochromes, hence minimizing the final compensation values, as a consequence the final experiment will be more “stable“. The photomultiplier voltages are adjusted to ensure maximum signal to noise for the antigen under investigation by the use of single fluorochrome, experiment specific stained cells. This will enable the visualization of non-specific antibody binding affects, and also where possible the percentage of the cells under very uncomplicated conditions, these percentages used to verify that under the polychromatic experiment conditions values remain the same. The single stained cells however may not have a high concentration of antigen, therefore we rely on antibody capture beads to “load” the experimental antibodies, singly onto antibody capture beads, this ensures that for all fluorochromes we have a very high fluorochrome signal, thus enabling accurate compensation values, avoiding errors associated with cells that may have low antigen concentration and consequently low signal and consequent inaccuracies when calculating compensation. It should be noted at this point that the verification of instrument photomultiplier linearity is a prerequisite, as is the verification of tandem fluorochrome integrity. With the optimization of photomultiplier voltages the compensation beads can now be run, either using the automatic compensation protocols on the instrument, or if preferred, adjusting the values manually aligning the negative and positive medians for the single discreet fluorochromes. Especially if the automated compensation protocol has been used, a verification step should be performed by appending each compensation bead into a single list mode file to ensure all beads are compensated, this can also be repeated using the single stained cells, although, in the cell scenario some of the antigens may be weak or absent. This step is imperative prior to running the tube where all antibodies are present so to ensure that compensation values are correct and there is no variance between the beads and cells i.e., they must both be correctly compensated. At this point the sample with all antibodies within the one tube is run, you have an immediate check, using previous single color tubes, to ensure that all percentages between the multicolor tube and the single color tubes correlate, also that there is no loss of intensity, this may sometimes occur due to dilution effects of other antisera when using large amounts of antisera. With careful scrutiny of the compensation values achieved you may be able to reduce compensation values by adjusting photomultiplier voltages but care must be taken not to compromise the sensitivity of the assay

